# Cytokines: The Future of Intranasal Vaccine Adjuvants

**DOI:** 10.1155/2011/289597

**Published:** 2011-07-31

**Authors:** Afton L. Thompson, Herman F. Staats

**Affiliations:** ^1^Department of Pathology, Duke University Medical Center, P.O. Box 3712, Durham, NC 27710, USA; ^2^Department of Immunology, Duke University Medical Center, Durham, NC 27710, USA; ^3^Human Vaccine Institute, Duke University Medical Center, Durham, NC 27710, USA

## Abstract

Due to its potential as an effective, needle-free route of immunization for use with subunit vaccines, nasal immunization continues to be evaluated as a route of immunization in both research and clinical studies. However, as with other vaccination routes, subunit vaccines often require the addition of adjuvants to induce potent immune responses. Unfortunately, many commonly used experimental vaccine adjuvants, such as cholera toxin and *E. coli* heat-labile toxin, are too toxic for use in humans. Because new adjuvants are needed, cytokines have been evaluated for their ability to provide effective adjuvant activity when delivered by the nasal route in both animal models and in limited human studies. It is the purpose of this paper to discuss the potential of cytokines as nasal vaccine adjuvants.

## 1. Introduction

The nasal route of immunization is an attractive route of immunization for several reasons. Perhaps most importantly, this route of immunization does not require needles, and the WHO is interested in needle-free methods of immunization because needle reuse can lead to disease transmission [1–5]. Intranasal immunization would also allow people with a needle phobia to receive vaccines that they may otherwise avoid, increasing the chances of establishing herd immunity [6–8]. Finally, nasal immunization has the ability to induce antigen-specific immunity in both the systemic and mucosal compartments (humoral and cellular), while parenteral immunization rarely induces mucosal immunity. The induction of antigen-specific mucosal immunity (including secretory IgA) may provide an added layer of protection against pathogens that first contact the host at a mucosal surface [9–11].

As with other routes of immunization, in the absence of potent vaccine antigens, such as live-attenuated organisms (virus, bacteria), vaccine adjuvants are required to boost the immune responses to less potent antigens, such as recombinant subunit proteins. Unfortunately, some of the most commonly used experimental mucosal vaccine adjuvants are not safe for use in humans. The most notable example is cholera toxin (CT). Although CT is considered to be the “gold-standard” mucosal vaccine adjuvant, amounts as low as 2.5 *μ*g delivered intranasally can induce antigen-specific IgE [12]. In addition, even mutant CT has been shown to redirect the codelivered vaccine antigen to the olfactory nerve and bulb of the brain in mice [13, 14]. *Escherichia coli* heat-labile toxin (LT) has also been shown to have negative side effects in humans, as both native and mutant LT (mLT) used as adjuvants were recently associated with the development of Bell's palsy following intranasal delivery in humans [15–17]. It is, therefore, evident that these toxin adjuvants exhibit safety concerns that will likely prevent their use in nasally delivered vaccines for humans. 

Since the early 1990s, many groups, including ours, have examined the ability of cytokines to serve as vaccine adjuvants due to their potent effects on the immune system. The use of cytokines as vaccine adjuvants has been previously reviewed [18–20], but we hope to offer a more in-depth and up-to-date review on the use of cytokines as nasal vaccine adjuvants. 

It is the purpose of this paper to highlight the immune responses that can be augmented by the use of cytokines as nasal vaccine adjuvants. We will discuss the ability of nasally delivered cytokine adjuvants to induce antigen-specific serum antibody, mucosal antibody, and cell-mediated immunity as well as to induce protection against a challenge with toxins or live bacteria/viruses. Although some of the studies discussed have focused on responses other than those highlighted, including cell trafficking and lymphocyte cell-surface molecule expression profiles, this paper will focus on vaccine-induced adaptive immune responses augmented by the use of cytokines as adjuvants for nasal vaccines. When possible, the adjuvant activity of cytokines will be highlighted by discussion of the antigen-specific immune responses induced in the absence of adjuvants and compared to the antigen-specific responses induced with the use of cytokine adjuvants. It is important to note that comparing antigen-specific immune responses between studies is sometimes difficult given that few use the same procedures for measuring and calculating the induced responses. For instance, ELISA values measured using a single sample dilution and reporting OD values are not as sensitive as endpoint titers, and it is therefore difficult to compare ELISA results between studies reporting endpoint titers and those reporting OD (Table 1).

## 2. IL-12

IL-12 is commonly known as a Th1 cytokine. It induces NK, T, and B cells to produce IFN*γ*, and it is the primary cytokine involved in Th1 differentiation [35]. 

### 2.1. Serum Antibody Production

When used as a nasal vaccine adjuvant, IL-12 has been shown by several groups to enhance serum antibody production, including IgM, IgG, and IgA, to a variety of antigens. However, IL-12 has been used at a variety of doses and dosing schedules with a variety of outcomes. For instance, Bradney et al. delivered 0.1 *μ*g IL-12 + 10 *μ*g HIV peptide to mice on days 0, 7, 14, and 28 but failed to see an increase in antipeptide IgG GMT compared to serum anti-HIV peptide IgG titers in mice immunized with peptide alone [31]. By contrast, Baca-Estrada et al. delivered 0.5 *μ*g IL-12 on days 0 and 21 with 0.8 *μ*g herpesvirus type 1 glycoprotein-D- (gD-)-containing liposomes and noted a significant increase in anti-gD serum IgG compared to immunization with gD-containing liposomes alone (~1 : 75,000 versus undetectable) [26]. The use of IL-12 in this nasal vaccine formulation also significantly increased serum bovine herpesvirus neutralizing antibody titers (approx. 1 : 650 versus undetectable in the absence of IL-12) [26]. Using a more complex dosing schedule that would not likely be relevant for human vaccine design, Boyaka et al. demonstrated increases in serum anti-TT IgG and IgA following vaccination with 1 *μ*g liposome-complexed IL-12 delivered on days 0, 3, 7, 10, 14, and 17 with 20 *μ*g TT delivered on days 0, 7, and 14 (approx. IgG titers of 1 : 2,097,152 versus 1 : 4,096 and IgA titers of 1 : 2,048 versus 1 : 32, resp.) [25]. In fact, the majority of studies delivering recombinant IL-12 (rIL-12) have used doses of 1 *μ*g, albeit with different total amounts and dosing schedules. Several studies have also examined the ability of IL-12 incorporated into a plasmid to induce immune responses [30]. Although it is difficult to compare doses between those studies and studies delivering rIL-12, all forms of IL-12 have consistently been shown to increase antigen-specific IgG2a and decrease antigen-specific IgG1 antibody production compared to antigen delivered alone [30, 36, 37], indicating that its coadministration with vaccine antigens induces a Th1 bias in the immune response. This is unsurprising given its in vivo role in immune response development. 

Very few studies delivering IL-12 with antigen have compared the IL-12-induced antibody response to the response induced by more common vaccine adjuvants, such as CT. Unfortunately, the few studies that have included CT as a control adjuvant did not observe significant increases in the antigen-specific antibody titer when using IL-12 as compared to antigen alone [30, 31, 38]. Although Okada et al. reported significant increases in anti-HIV IgG2a following IL-12 + antigen immunization, they only compared the antigen + IL-12-induced anti-HIV total IgG responses to those induced by antigen + 10 *μ*g CT; neither group induced a significant increase in anti-HIV IgG when compared to serum antibody titers measured in mice immunized with antigen alone [30]. Albu et al. also compared the antigen-specific IgG response induced by vaccination with antigen + IL-12 or CTB [38], and they also failed to detect an increase in antigen-specific antibody with either adjuvant. It is, therefore, difficult to determine the significance of IL-12-induced antigen-specific serum antibody production with respect to other well-known adjuvants.

IL-12 has also frequently been used in combination with other adjuvants. Although Brandtzaeg failed to observe adjuvant activity when IL-12 was administered nasally to mice with an HIV peptide antigen, they did observe an increase in serum antipeptide IgG when mice were nasally immunized with HIV peptide combined with 0.1 *μ*g rIL-12 mixed with IL-1 and IL-18 [10]. Similarly, Mutsch et al. demonstrated that 0.1 *μ*g rIL-12 incorporated into a vaccine already containing 1 *μ*g CT could enhance anti-TT IgG2a production and inhibit anti-TT IgE [17]. These observations suggest that low-dose rIL-12 may be useful to enhance the adjuvant activity of other adjuvants while simultaneously enhancing IgG2a/Th1 responses.

### 2.2. Mucosal Antibody Production

Several studies have demonstrated the ability of rIL-12 to induce mucosal IgA production to a variety of codelivered antigens. However, unlike many of the studies that examined serum antibody production, a number of studies examining mucosal antibody production with rIL-12 have used alternate dosing schedules, with IL-12 delivery given both with the vaccine and then at various time points following vaccination. For instance, Boyaka et al. delivered 1 *μ*g liposome-complexed IL-12 to mice on days 0, 3, 7, 10, 14, and 17 with an antigen delivery schedule of 0, 7, and 14 [25]. Using this schedule, they induced the production of fecal (1 : 512), vaginal (1 : 23), and salivary (1 : 64) anti-TT IgA that was significantly increased when compared to anti-TT IgA responses induced by immunization with TT alone (~1 : 4). They also noted significant IL-12-dependent increases in IgA AFCs in the lungs, CLNs, and lamina propria lymphocytes (19, 15, and 934 versus 1, 1, and 23 AFC/10^6^ cells, resp.). Similarly, Lynch et al. delivered 1 *μ*g IL-12 on days 0, 1, 2, and 3 with pneumococcal polysaccharide (PPS3) delivery on day 0. Using this dosing schedule, they noted a significant increase in BAL anti-PPS3 IgA, but this was reported only as an OD increase at an undefined BAL dilution [37]. Interestingly, using a similar dosing schedule to Lynch et al., with antigen delivery on day 0 and IL-12 delivery on days 0, 1, 2, and 3, Arulanandam and Metzger observed a significant decrease in fecal anti-OVA IgA production; this was also reported only as a change in OD at an undefined fecal extract dilution [36]. Baca-Estrada et al. used a more typical dosing schedule of 0 and 21 days with antigen-containing liposomes and free IL-12; although absolute increases in lung antigen-specific IgA were noted (Ag alone, 1 : 66; Ag + free IL-12, 1 : 577), the increases were not significant due to large amounts of animal-to-animal variation [26]. Unfortunately, the different methods of measuring the immune response between the studies makes it difficult to compare their results because Boyaka et al. and Baca-Estrada et al. were the only groups to calculate endpoint titers for mucosal antibody responses following vaccination with antigen + IL-12 only. Nonetheless, it does appear that IL-12 has the potential to increase mucosal IgA production, but more studies need to be carried out to confirm this, including studies comparing IL-12 to more well-known mucosal adjuvants.

### 2.3. Cell-Mediated Immunity

As a Th1-associated cytokine, IL-12 has been evaluated for its ability to induce CD8+ cytotoxic T lymphocytes as well as delayed-type hypersensitivity reactions. The groups that have examined the ability of IL-12 to induce these responses have used three different forms of IL-12, including recombinant protein [39], plasmid expressed [30, 40], and bacterium expressed [41]. Using 0.1 *μ*g rIL-12 as an adjuvant coadministered with an HIV peptide immunogen, we observed no significant increase in peptide-specific lysis compared to mice immunized with antigen alone at a variety of antigen doses at an E : T ratio of 25 : 1 [39]. In this same study, CT used as a positive control adjuvant induced ~40–60% peptide-specific lysis. We also failed to see an increase in the number of peptide-specific IFN*γ*-secreting CD8+ T cells. By contrast, when Bermúdez-Humarán et al. immunized mice with a recombinant lactococci secreting the HPV E7 protein and IL-12, cell-mediated cytotoxicity was significantly increased compared to cytotoxic T lymphocyte (CTL) responses induced by vaccination with a recombinant lactococci secreting only E7 (~15% versus 5%, resp.) at an E : T ratio of 30 : 1 [41]. Using the same formulation, they also observed significant increases in the numbers of antigen-specific IFN*γ*-secreting CD4+ and CD8+ T cells over those induced by antigen alone (CD8 : 200 versus 100 per 10^4^ cells, CD4: 225 versus 50 per 10^4^ cells). Considering that the % lysis we reported following immunization with peptide alone was near 15%, it is possible that the differences between our study and Bermúdez-Humarán's lay in the type of antigen (protein versus peptide) or the potency of the antigen. 

Codelivering 10 *μ*g of a plasmid expressing IL-12 and 2 *μ*g each of two HIV protein-expressing plasmids, Xin et al. demonstrated significantly increased antigen-specific lysis compared to that induced by immunization with antigen alone at an E : T ratio of 20 : 1 (~45% versus <20%, resp.) following only one vaccination [40]. When Okada et al. included 2 *μ*g of pIL-12 in a vaccine formulation with liposomes and plasmid-expressed antigen delivered three times, they also observed an increase in the antigen-specific lysis at an E : T ratio of 25 : 1 when compared to antigen plus liposomes [30]. However, they did not compare the ability of IL-12 to increase specific lysis in the absence of liposomes. It is interesting to note that although we did not observe a significant increase in antigen-specific lysis following three immunizations with antigen + IL-12, adding an additional boost with antigen alone (~10% versus <5%, resp.) resulted in a significant increase in antigen-specific lysis compared to antigen alone delivered four times (at an E : T ratio of 20 : 1). Given that we delivered a total of 0.3 *μ*g IL-12 over the three immunizations, it is likely that we also delivered the lowest dose of IL-12 of the studies reported here. Had the IL-12 dose been optimized and increased into the ranges used by other studies to induce serum antibody production, it is possible that a stronger effect of IL-12 on specific lysis would have been seen. However, three of the four studies discussed here did observe significant increases compared to antigen alone (or plus liposomes) when IL-12 was added to the vaccine formulation.

### 2.4. Protection against Challenge

The ability of IL-12 to augment vaccine-induced immune responses and enhance protection against infectious challenge has been evaluated using both viral and bacterial challenge models. Two weeks following intranasal immunizations on days 0, 7, and 14 with a TT vaccine, Boyaka et al. challenged mice with 100 MLDs (minimum lethal doses) of TT. All mice that received the vaccine formulation with TT + liposome-complexed IL-12 (1 *μ*g delivered on days 0, 3, 7, 10, 14, and 17) survived the challenge; none of the mice receiving TT alone survived [25]. Following one vaccination with a pneumococcal conjugate vaccine (day 0) and 1 *μ*g IL-12 on days 0, 1, 2, and 3, Lynch et al. challenged mice with 5 × 10^4^ CFU type 3 pneumococcal bacteria. Although the vaccination regimen did not induce complete protection against challenge, nearly 80% of mice that received the vaccine and IL-12 survived the challenge, compared to 40% survival of those that received the vaccine only [37]. Using a different approach, Parker et al. evaluated the ability of vaccination with IL-12-expressing HSV-1 to protect against systemic challenge with 100 LD_90_ of a highly pathogenic HSV strain [42]. Mice immunized with as few as 100 pfu HSV/IL-12 had significantly greater survival than sham-immunized mice (0/29). At this dose, 4/19 mice survived compared to 0/19 mice immunized with the parent virus. As the immunization dose increased, so did survival following challenge, with 92% and 100% of mice immunized with 10,000 and 100,000 pfu surviving challenge, compared to 30% and 80% of mice immunized with the parent strain. Although only a few studies have evaluated the ability of IL-12 to induce protective immunity, it does appear to be effective in this regard.

## 3. IL-2

IL-2 also involved in T-cell proliferation and the induction of T regulatory responses [43]. As such, it has typically been investigated for its ability to induce CTLs (see below), but its ability to induce serum antibody production has been examined by a small number of groups. 

### 3.1. Serum Antibody Production

In two separate studies, Xin et al. demonstrated that a single administration of plasmid-incorporated IL-2 to mice induced significant decreases in serum anti-HIV IgG1 and increases in serum anti-HIV IgG2a (16- and 10-fold, resp., compared to antigen alone) following codelivery with two other plasmids expressing HIV proteins [40, 44]. Steidler et al. took a different approach and engineered a recombinant *L. lactis* that coexpressed IL-2 and tetanus toxin fragment C (TTFC) [23]. Following nasal inoculation on days 0, 14, and 28 with 10^9^ bacteria, the IL-2-expressing bacterium induced significant increases in serum anti-TTFC IgG on days 24, 35, and 80 postprimary immunization (approx. 10-, 10-, and 5-fold higher than TTFC-expressing bacteria alone).

Macaques have also been used to examine the effectiveness of IL-2 as a nasal vaccine adjuvant [45]. Following four nasal immunizations with 1.5 mg DNA containing a mutated SIVmac239 genome and IL-2, macaques were challenged rectally, and serum antibody responses were then monitored. At 6–8 weeks after the challenge, animals immunized with IL-2 had significantly greater gp120- and gp41-specific IgG (~1 : 50,000) than those given no incorporated cytokine (~1 : 1,000) or incorporated IL-12 (~1 : 5,000); by 12–16 weeks, animals receiving IL-2 had also increased anti-SIV IgG compared to the other immunization groups (~1 : 50,000 versus ~1 : 8,000) [45]. This study also evaluated the ability of IL-2 to augment virus-neutralizing antibodies and demonstrated a nearly 50-fold increase from ~1 : 100 in antigen-alone immunized macaques to ~1 : 50,000 in macaques immunized with the vaccine formulated with IL-2. Although this study used a slightly different approach, examining antibody titers after challenge, the results indicate that IL-2 is effective at inducing serum antigen-specific and neutralizing antibody production in nonhuman primates and demonstrates the efficacy of IL-2 used as a nasal vaccine adjuvant.

### 3.2. Mucosal Antibody Production

Of the numerous studies that have examined intranasal delivery of IL-2, only a few have examined mucosal antibody production. Two such studies were performed in mice by Abraham and Shah [46] and Ferko et al. [24] using very different delivery methods. While Abraham and Shah used a liposomal formulation to deliver 25 *μ*g levan (a bacterial polysaccharide) and 0.02–0.2 *μ*g IL-2, Ferko et al. delivered 2 × 10^5^ PFU of an IL-2-expressing influenza virus (*ca* Sing-IL-2). Unlike many studies involving other nasally delivered cytokine adjuvants, Abraham and Shah noted an increase in the total number of pulmonary antibody-secreting plasma cells (ASCs) (from 3,712 to ~7,480 per set of lungs) in addition to an increase in the number of levan-specific ASCs (undetectable to ~330 per set of lungs), of which 90% were antilevan IgA ASCs. The amount of antilevan IgA in lung lavages was also significantly increased at both 0.02 and 0.2 *μ*g IL-2, but these increases were only presented as significant increases in OD at a 1/3 dilution. Following delivery of *ca* Sing-IL-2, Ferko et al. also demonstrated a 3.5-fold increase in the production of nasal virus-specific IgA (1 : 50 for *ca* Sing versus 1 : 200 for *ca* Sing-IL-2) and a 2-fold increase in vaginal virus-specific IgA compared to *ca* Sing (1 : 200 and 1 : 400, resp.). 

In addition to the studies in mice, one group has also examined the impact of IL-2 incorporation into an avian influenza virus vaccine in chickens [47]. In this study, chickens were immunized twice (days 0 and 14) with approximately 105 EID_50_ H5N2 with or without 50 *μ*g IL-2. In the lung and trachea, the total number of IgA and IgG ASCs increased significantly at weeks 3, 5, and 7 following vaccination with virus + IL-2 compared to vaccination with virus alone. However, this study did not examine the impact of vaccination on virus-specific ASCs or IgA, and the number of IgA-secreting cells was counted in fixed tissue sections instead of in an ELISPOT assay. In contrast to many of the studies using a mouse model, however, Xiawen et al. compared the adjuvant activity of IL-2 to an equivalent dose of CpG. CpG and IL-2 induced statistically equivalent amounts of IgA-secreting cells at all time points examined. Although IL-2 induction of mucosal IgA responses has not been often examined, studies thus far are encouraging.

### 3.3. Cell-Mediated Immunity

Using an influenza virus expressing IL-2, Ferko et al. induced a twofold increase in the number of antigen-specific IFN-*γ*-secreting cells in both the spleen and lymph nodes of immunized mice 10 days after the primary immunization and a nearly fourfold increase in the mediastinal lymph nodes 4 weeks after the primary immunization compared to IFN*γ* responses induced by immunization with virus alone [24]. In addition, two studies by Xin et al. demonstrated significantly increased target cell lysis following delivery of 10 *μ*g pIL-2 at E : T ratios of both 20 : 1 and 80 : 1 (increases of ~40% and ~30% compared to lysis induced by immunization with plasmid-expressed antigen alone) [40, 44]. They also demonstrated significant increases in DTH following codelivery of 1 or 10 *μ*g pIL-2 compared to antigen alone (14.2 and 16.7 versus 11.1 × 10^−2^ mm, resp.).

### 3.4. Protection against Challenge

The ability of IL-2 to induce protective immune responses when intranasally codelivered with a vaccine has been evaluated in both mouse and monkey models. Using a mouse model of influenza infection, Ferko et al. evaluated the ability of immunization with an IL-2-expressing influenza virus to protect mice against challenge with 2 × 10^5^ pfu of the Sing-WT virus [24]. The addition of IL-2 to the vaccine provided 100% protection while immunization with virus alone provided only 57% protection. *ca *Sing-IL-2 also decreased the viral titer in the lungs from 620 (*ca *Sing) to <86 TCID_50_/g tissue. Similarly, in a bacterial challenge model, Abraham and Shah increased survival following challenge with 5 × 10^7^ cfu *P. aeruginosa* from ~10% in those receiving antigen-containing liposomes to 45% in those receiving liposomes-containing antigen and IL-2 [46].

In a macaque model of SIV infection, Bertley et al. used CD4+ T cell counts as a marker of disease progression following rectal challenge with 10 50% animal infectious doses (ID_50_) of pathogenic SHIV89.6P virus [45]. Four nasal immunizations with 1.5 mg DNA containing a mutated SIVmac239 genome and IL-2 did not significantly increase the CD4+ T cell count compared to those receiving the DNA vaccine alone. The addition of IL-2 did, however, provide greater protection than the vaccine formulation that included IL-12, as 5/5 animals immunized with antigen and IL-2 survived the challenge, while only 1/5 animals immunized with antigen and IL-12 survived. Interestingly, 4/5 animals receiving the DNA vaccine alone and 1/5 of the nonimmunized animals survived; two of the macaques receiving the DNA vaccine alone seroconverted, and viremia was never detected. Although the DNA vaccine alone was effective at protecting some of the animals, IL-2 does appear to have provided some level of increased protection in this model as well.

## 4. IL-15

IL-15 is known to share several overlapping activities with IL-2, which is likely due to their extremely high homology and their structural similarities [48]. In addition to its ability to promote both NK and T-cell development and proliferation [48], IL-15 is known to augment B-cell antibody production, and so its incorporation into nasal vaccines could be beneficial [49]. 

### 4.1. Serum Antibody Production

Two studies investigating the ability of IL-15 to provide nasal vaccine adjuvant activity have shown similar results. Toka et al. delivered 50 *μ*g IL-15 and 50 *μ*g of DNA encoding herpesvirus glycoprotein B (gB) to mice on days 0 and 21. At 64 days after the boost, the incorporation of IL-15 did not significantly increase serum anti-gB IgG titers compared to gBDNA alone (1 : 22,000 versus 1 : 19,061, resp.) [27]. When Xin et al. delivered one dose of 2 *μ*g pCMV160IIIB/REV + 10–50 *μ*g IL-15 plasmid, although serum anti-HIV IgG2a titers were significantly increased compared to antigen alone, the real change in titer was small (1 : 32 versus 1 : 11), and serum anti-HIV IgG titers were unchanged [40]. Given that Xin et al. did see an increase (albeit small) in serum anti-HIV IgG2a, it is possible that Toka and Rouse could have seen increased anti-gB IgG2a titers had they been investigated. However, it is difficult to compare the two studies given the different forms of IL-15 and the different dosing schedules.

### 4.2. Mucosal Antibody Production

Two studies have evaluated IL-15 for its ability to augment vaccine-induced mucosal IgA, although they provide conflicting results. Following the administration of 50 *μ*g IL-15 DNA with 50 *μ*g gBDNA, Toka and Rouse noted a fourfold increase in vaginal anti-gB IgA when compared to the vaginal anti-gB IgA response induced by immunization with gBDNA alone. The elevated vaginal IgA response persisted for 300 days [27]. By contrast, using 2 *μ*g pCMV160IIIB/REV plasmid and 10 *μ*g plasmid-encoded IL-15, Xin et al. failed to see an increase in fecal anti-HIV IgA. There are several possible reasons for these conflicting results. Although both groups immunized with plasmid-encoded IL-15, it is difficult to compare the actual delivered dose of cytokine. In addition, Toka and Rouse investigated vaginal IgA production, while Xin et al. evaluated fecal IgA production. Although nasal immunization can induce the production of fecal IgA, it more commonly induces IgA production in the respiratory and reproductive tracts [50, 51]. It is, therefore, not surprising that Xin et al. failed to see an increase in anti-HIV fecal IgA with the use of IL-15 as an adjuvant when compared to mice immunized with antigen alone. Nonetheless, studies examining the ability of IL-15 to induce mucosal antibody production at more sites with a larger number of antigens are required before it can be said definitively that IL-15 is a potent inducer of mucosal IgA production.

### 4.3. Cell-Mediated Immunity

Following delivery of plasmids expressing antigen and IL-15 (10 or 50 *μ*g), Xin et al. demonstrated significant increases in antigen-specific lysis at E : T ratios of 80 : 1 (~70% lysis), 20 : 1 (40% and 35% lysis, resp.), and 5 : 1 (30% and 25% lysis, resp.) when compared to the specific lysis induced by immunization with only the antigen-expressing plasmid (~30%, 20%, and 10% at 80 : 1, 20 : 1, and 5 : 1, resp.). With the exception of the lowest E : T ratio, their results were similar to those seen when they delivered the same doses of plasmids expressing IL-12 or IL-2 [40]. The delivery of 10 *μ*g pIL-15 also significantly increased the DTH response 14 days after immunization compared to DTH responses induced by immunization with antigen alone (14 versus 10 ×10^−2^ mm, resp.). Toka and Rouse evaluated the ability of codelivered pIL-15 to augment the number of antigen-specific IFN*γ*-secreting CD8+ T cells in the spleen at both 7 and 60 days after a booster immunization and observed 3- and 3.6-fold increases, respectively, in antigen-specific CD8+ responses when compared to those induced by immunization with antigen alone [27]. They also demonstrated an increased number of tetramer-positive antigen-specific CD8+ T cells at 300 days after the boost (1.67% versus 0.69% of gated cells), but they did not observe an increase in peptide-specific lysis compared to antigen delivery alone.

### 4.4. Protection against Challenge

Toka and Rouse vaginally infected mice with 1 × 10^7^ pfu HSV 65 days after their second and final immunization with a DNA vaccine. Codelivery of pIL-15 increased survival from 2/6 mice (DNA vaccine alone) to 5/6 mice and decreased the viral titer from 1 × 10^5^ pfu/mL to 1 × 10^2.5^ pfu/mL, though it is unclear in what tissue/fluid the viral titer was measured.

## 5. Type I IFN

Although type I IFNs are frequently thought of as primarily involved in antiviral immune responses, they have several other roles as well, including T-cell proliferation, NK cell activation, and cytokine induction [52]. Interestingly, previous studies have linked the adjuvant activities of a TLR9 agonist (IC31) [53] and complete Freund's adjuvant [54] to the presence of IFNAR1.

### 5.1. Serum Antibody Production

Type I IFN nasal vaccine adjuvant activity has primarily been investigated when codelivered with influenza antigens [15, 33, 34]. Using 5 or 11.75 *μ*g of hemagglutinin, respectively, Bracci et al. [33] and Proietti et al. [34] demonstrated increased anti-HA serum IgG and IgG2a two weeks after the second of two vaccinations compared to HA delivered alone. Bracci et al. demonstrated serum anti-HA IgG increases from ~1 : 200 to ~1 : 35,000 and serum IgG2a from undetectable to ~1 : 25,000 when using 4 kU IFN as a nasal vaccine adjuvant. Likewise, Proietti et al. observed increases in serum anti-HA IgG from ~1 : 10,000 to ~1 : 316,000 and anti-HA IgG2a from ~1 : 63 to ~1 : 16,000. Although the dose of IFN delivered by Proietti in this experiment is unclear, it is evident that type I IFN can increase serum HA-specific antibody titers in mice. Unlike many of the other studies performed with nasally delivered cytokine adjuvants, Proietti et al. also compared the abilities of type I IFN and MF59 to augment serum antibody production. Using a 1 : 1 (v:v) ratio of MF59 : antigen, MF59 induced greater amounts of anti-HA IgG1 than type I IFN (1 : 30,000 versus 1 : 10,000, resp.), but it induced less anti-HA IgG2a and IgA (1 : 20,000 versus 1 : 60,000 and 1 : 20,000 versus 1 : 130,000, resp.).

Couch et al. evaluated the effectiveness of type I IFN as an adjuvant to an influenza vaccine in both mice and humans [55]. Although 10 kU IFN provided adjuvant activity comparable to 5 *μ*g CTB or 7.5 *μ*g MPL in mice given 0.3 *μ*g HA on days 0, 1, 28, and 29 (GMT neutAb: 1 : 49–1 : 64 versus 1 : 5 for HA alone), it did not provide significant adjuvant activity in humans when given at 10 million units. However, in the human study, the vaccine was only administered intranasally once, and it used a different vaccine antigen (HA versus inactivated trivalent influenza vaccine). As the inactivated trivalent influenza vaccine alone was immunogenic in humans and resulted in elevated serum anti-HA antibody responses after nasal immunization, it is possible that the superior immunogenicity of the inactivated vaccine masked the adjuvant activity of IFN. Within the mouse model, type I IFN does appear to induce significant increases in antigen-specific antibody titers, but more in-depth studies in larger animal models may indicate whether type I IFN would be an effective intranasal adjuvant in humans.

### 5.2. Mucosal Antibody Production

The ability of type I IFNs to augment mucosal antibody production has not been frequently evaluated, but the results are encouraging. When vaccinating mice with 11.75 *μ*g HA and 40 kU IFN, Proietti et al. observed a 10-fold increase in BAL anti-HA IgA compared to anti-HA IgA induced by immunization with antigen alone (1 : 6,300 and 1 : 630, resp.). In anesthetized mice, Bracci et al. did not observe significant increases in BAL anti-HA IgA following two immunizations with 5 *μ*g HA + 40 kU IFN (<1 : 10) compared to antigen alone, but they did detect a significant increase following the same dosing regimen in unanesthetized mice (1 : 40 versus undetectable for HA alone). Using 4 and 40 kU IFN + HA, nasal lavage anti-HA IgA was significantly increased above antigen alone-induced anti-HA IgA in both anesthetized (1 : 300 versus 1 : 100) and unanesthetized (1 : 550 versus undetectable) mice, respectively, but the increase was greater in the latter group. The effects of anesthesia on nasal vaccine-induced antigen-specific immunity have been previously examined [21, 56]. Although these studies have demonstrated that sedation typically correlates with increased antigen-specific immunity in serum, Janakova et al. did not observe an impact on antigen-specific saliva or fecal IgA production [56]. The results reported by Bracci et al. are therefore interesting but could be explained, perhaps, by the sites at which the mucosal responses were evaluated. 

Couch et al. also investigated mucosal antibody production following vaccination of mice and humans. In mice, although they evaluated the H1N1 vaccine alone or combined with CTB, MPL, or type I IFN, only vaccination with HA and MPL induced significant increases in lung antigen-specific antibody and no group induced significant increases in nasal secretion antibody production compared to HA alone. Their results were similar in humans, with no significant increases in antigen-specific nasal secretion IgG or IgA when IFN was included in the vaccine compared to vaccine alone.

### 5.3. Protection against Challenge

Although type I IFNs have been evaluated for their ability to protect against challenge, they have only been evaluated for their ability to protect against challenge with influenza virus. Following challenge with 10 LD_50_ of influenza virus, both 4 and 40 kU IFN were found to provide 100% protection compared to 40% and 60% protection after immunization with vaccine alone ([33] and [34], resp.). In another study, Couch et al. evaluated the ability of four intranasal vaccinations with 10 kU IFN and 0.3 *μ*g HA to increase mouse survival following challenge with 100 ID_50_ of influenza virus [55]. Inclusion of IFN in the vaccine formulation decreased the viral lung titer from 6.2 log_10_/lung to 2.0 log_10_/lung, which was similar to the other two tested adjuvants, CTB (2.6 log_10_/lung) and MPL (2.2 log_10_/lung). These studies suggest that type I IFNs can be effective at increasing vaccine-induced protection against influenza viruses.

## 6. MIP-1***α***/MIP-1***β***

Like many cytokines, the MIP-1 family of chemotactic cytokines are known best for their roles in innate immune responses, including the recruitment of proinflammatory cells [57]. However, they also have the ability to modulate Th differentiation, as MIP-1*α* has been shown to promote Th1 responses [57], and blocking MIP-1*α* has been shown to reduce Th1 responses to *Cryptococcus neoformans* infection [58].

### 6.1. Serum Antibody Production

The ability of MIP-1*α* to induce serum antibodies has been investigated using at least two different antigens: ovalbumin and HIV-REV [32, 59]. Using plasmid-expressed MIP-1*α*, Lu et al. demonstrated increased serum anti-HIV IgG, with significantly decreased IgG1 and significantly increased IgG2a compared to the pCMV160IIIB/REV plasmid alone four weeks following a single administration to mice [59]. The absolute increase in titer was low, however, with serum anti-HIV IgG increasing from 1 : 147 to 1 : 416; anti-HIV IgG1 and IgG2a titers were only reported as ODs at an unspecified dilution. Recombinant MIP-1*α* coadministered with ovalbumin has also been shown to induce serum antibody production in mice [32]. A dose of 1 *μ*g rMIP-1*α* significantly increased serum anti-OVA IgM and IgG titers above those induced by OVA alone, though the largest increase was in anti-OVA IgG, from approximately 1 : 128 to 1 : 131,072. This dose of MIP-1*α* significantly increased all anti-OVA IgG subclasses over those induced by OVA alone, with the greatest increases in IgG1 (~1 : 128 versus ~1 : 131,072) and IgG2b (~1 : 64 versus ~1 : 8,192), followed by IgG2a (<1 : 32 versus ~1 : 1,024) and IgG3 (<1 : 32 versus ~1 : 128). Although the study by Lu et al. did not observe drastic increases in serum anti-HIV IgG, the study by Lillard et al. [32] indicates that MIP-1*α* shows promise as a vaccine adjuvant for increasing serum antigen-specific antibody production.

In contrast to MIP-1*α*, Lillard et al. saw a different profile of antibody responses when they used MIP-1*β* as an adjuvant. MIP-1*β* induced levels of serum anti-OVA IgA, IgG, and IgE by day 21 that were significantly greater than those induced by OVA alone (1 : 128, 1 : 32,768, and 1 : 512, resp.) [32]. Interestingly, MIP-1*β* induced smaller increases in serum anti-OVA IgG1 and IgG2b than MIP-1*α* (1 : 32,768 and 1 : 1,024, resp.), but it did not induce any increases in anti-OVA IgG2a or IgG3.

### 6.2. Mucosal Antibody Production

Of the two studies that have examined nasal delivery of MIP-1*α* as a vaccine adjuvant, both have investigated its ability to augment mucosal antibody responses. Lu et al. observed a significant increase in fecal anti-HIV IgA following intranasal administration of an HIV plasmid plus 10 *μ*g of pMIP-1*α* compared to fecal anti-HIV IgA responses induced by immunization with HIV plasmid alone (1 : 294 versus 1 : 91, resp.) [59]. Similarly, Lillard et al. demonstrated a significant increase in fecal (1 : 77), vaginal (1 : 23), and nasal (1 : 9) anti-OVA IgA in mice given 75 *μ*g OVA + 1 *μ*g rMIP-1*α* compared to IgA responses induced in mice administered OVA alone (<1 : 2), though titers of 1 : 23 and 1 : 9 are not likely to be biologically significant [32]. Lillard et al. also evaluated the number of ASCs in the nasal tract, lung, and intestinal lamina propria (ILP). The inclusion of rMIP-1*α* in the vaccine formulation significantly increased the number of nasal OVA-specific IgM, IgG, and IgA ASCs compared to OVA alone to 200–250 ASCs/10^6^ cells from 100, 50, and 50 ASCs/10^6^ cells, respectively. Lung OVA-specific IgM (~250 versus <50 ASCs/10^6^ cells), IgG (~100 versus <50 ASCs/10^6^ cells) and IgA (~50 versus <10 ASCs/10^6^ cells) ASCs as well as ILP OVA-specific IgA ASCs (~350 versus undetectable) were also increased by vaccination with OVA + rMIP-1*α* compared to OVA alone. It, therefore, appears that MIP-1*α* can effectively induce mucosal IgA responses to codelivered antigen in mice.

In the same study, Lillard et al. also compared rMIP-1*α* to rMIP-1*β* [32]. Including 1 *μ*g MIP-1*β* in the formulation induced nearly twice the amount of anti-OVA IgA production as MIP-1*α* in feces, vaginal washes, and nasal washes (1 : 152, 1 : 61, and 1 : 15, resp., for MIP-1*β*) [32]. However, the effect on the numbers of OVA-specific AFCs was not as great in comparison to MIP-1*α*. In the nasal tract, lungs, and spleen, the two proteins induced similar amounts of OVA-specific IgM-, IgG-, and IgA-secreting cells, but MIP-1*β* induced a greater number of IgA-secreting cells in the ILP (MIP-1*α*: ~350/10^6^ AFCs, MIP-1*β*: ~600/10^6^ AFCs).

### 6.3. Cell-Mediated Immunity

Both MIP-1*α* and MIP-1*β* proteins have been shown to augment cell-mediated immunity when used as nasal vaccine adjuvants. Codelivering 1, 10, or 50 *μ*g pMIP-1*α* with a DNA vaccine significantly increased the DTH response two weeks after immunization (13.6, 15.6, and 11.5 × 10^−2^ mm) when compared to DTH responses induced by immunization with the DNA vaccine alone (9.2 × 10^−2^ mm) [59]. Lu et al. were also able to demonstrate that MIP-1*α* induced a significant increase in antigen-specific lysis using splenocytes at E : T ratios of 80 : 1 and 20 : 1 (40% and 25%) when compared to responses induced by immunization with antigen alone (<10% lysis). Following the delivery of 1 *μ*g rMIP-1*α* or 1 *μ*g rMIP-1*β* three times with 75 *μ*g OVA, Lillard et al. demonstrated significant increases in CD4+ T-cell proliferation in the spleen, Peyer's patches (PPs), lower respiratory tract, and cervical lymph nodes one week after the final immunization (10–12% proliferating cells) when compared to proliferative responses induced by immunization with antigen alone (<2%). Although Lillard et al. and Lu et al. saw similar increases in mucosal and systemic CTL for MIP-1*α*, the inclusion of MIP-1*β* did not significantly enhance the induction of mucosal or systemic CD8+ CTLs [32]. MIP-1*α* increased antigen-specific target cell lysis by CD8+ cells isolated from the spleen, CLNs, and PPs to near 50% at an E : T ratio of 50 : 1, compared to 0% specific lysis in mice immunized with antigen alone. By contrast, MIP-1*β* increased target cell lysis to only 10% at 50 : 1 in the spleen and failed to increase it at the other sites [32]. Similar to the other immune responses examined, these two proteins appear to have differing effects on cell-mediated immunity, with MIP-1*α* perhaps being more effective adjuvant for inducing these responses. However, with only one study examining this effect of MIP-1*β*, it is impossible to draw concrete conclusions, and the two proteins should be evaluated more indepth in the future.

## 7. GM-CSF

As a stimulating factor, GM-CSF induces the proliferation of macrophage, erythroid, granulocyte, eosinophil, megakaryocyte, and multipotent progenitors [60]. However, its receptor is also expressed on monocytes, macrophages, granulocytes, and lymphocytes, and GM-CSF is required for the in vitro differentiation of DCs [60].

### 7.1. Serum Antibody Production

Several studies have demonstrated that GM-CSF is able to increase antigen-specific serum antibody production following intranasal vaccination. Okada et al. delivered 2 *μ*g of a DNA vaccine with 2 *μ*g of plasmid GM-CSF (pGM-CSF) and liposomes on days 0, 7, and 21 [30]. Using this procedure, the use of pGM-CSF significantly increased anti-HIV serum IgG production on day 28, with a fourfold increase in serum titer compared to antigen plus liposomes alone (1 : 16,000 versus 1 : 4,000). This group also compared the ability of CT to augment immune responses when coadministered with the DNA vaccine in the absence of the liposomes and found no significant increase compared to antigen plus liposomes. Unfortunately, because CT was not administered with the liposome formulation, it is impossible to compare the adjuvant activities of CT and GM-CSF in this study. Okada did demonstrate, however, that pGM-CSF had a superior capacity to increase anti-HIV IgG compared to an identical dose of pIL-12 [30]. Using a different approach, Kim et al. evaluated the ability of GM-CSF to augment immune responses when expressed by an adenoviral vector [61]. In this study, mice were immunized four times on a triweekly basis with 1 × 10^8^ pfu of adenovirus expressing amyloid *β* (a*β*) with or without 1 × 10^8^ pfu of an adenoviral vector expressing GM-CSF. Mice immunized with the GM-CSF-expressing adenovirus had increased anti-a*β* serum IgG, IgG1, and IgG2b (~20 *μ*g/mL) compared to mice receiving the a*β*-expressing adenovirus alone (negligible). They also reported small (<5 *μ*g/mL) increases in anti-a*β* IgG2a and no increases in anti-a*β* IgM compared to the a*β*-expressing adenovirus alone. In contrast to these two studies, which both used vectors expressing GM-CSF, Bradney et al. delivered 4 *μ*g rGM-CSF with 10 *μ*g of an HIV peptide on days 0, 7, 14, and 28. GM-CSF significantly increased serum antipeptide IgG responses (1 : 10,000) compared to antigen alone (<1 : 10), and the increase was comparable to that induced by 1 *μ*g CT (1 : 10,000). Taken together, these mouse studies indicate that GM-CSF is an effective adjuvant for inducing antigen-specific serum immune responses when delivered nasally.

One group evaluated the ability of GM-CSF to augment neutralizing antibody titers [62]. However, following immunization with 5 × 10^5^ pfu of an attenuated vesicular stomatitis virus expressing GM-CSF, no increase in VSV-neutralizing antibody titers was seen compared to a similar virus expressing EGFP (3,413 versus 2,560, resp.). However, as this is the only study known to us that has examined neutralizing antibody production following intranasal immunization with GM-CSF, it is difficult to say whether a different delivery method (e.g., recombinant protein) would result in neutralizing antibody production.

### 7.2. Mucosal Antibody Production

Okada et al. delivered 2 *μ*g of a DNA vaccine with 2 *μ*g of pGM-CSF and liposomes on days 0, 7, and 21 [30]. Using this procedure, they demonstrated a significant increase in anti-HIV fecal IgA production on day 28 compared to fecal IgA responses induced by immunization with antigen plus liposomes (1 : 16,000 versus 1 : 5,700). When they compared the ability of CT to augment fecal IgA production after immunization with DNA vaccine in the absence of the liposomes, they found no significant increase compared to antigen plus liposomes. Unfortunately, because CT was not administered with the liposome formulation, it is impossible to compare the adjuvant activities of CT and GM-CSF in this study.

### 7.3. Cell-Mediated Immunity

Following two intranasal administrations of 0.2 *μ*g DNA vaccine and 0.2 *μ*g pGM-CSF, Okada et al. observed no significant increase in antigen-specific footpad swelling compared to footpad swelling in mice immunized with DNA vaccine alone (15.8 versus 14.3 × 10^−2^ mm) [30]. This study also evaluated the ability of pGM-CSF to enhance antigen-specific target cell lysis when added to a vaccine formulation containing pIL-12 + antigen. The addition of pGM-CSF significantly increased specific lysis at E : T ratios of 25 : 1 and 5 : 1 (~50% and 28%, resp.) compared to the lytic responses measured after immunization with the pIL-12 + antigen formulation (~40% and 18%, resp.). Unfortunately, the ability of pGM-CSF by itself to increase immune responses above those induced by antigen alone was not evaluated. We determined that rGM-CSF did not provide adjuvant activity for the induction of peptide-specific CTL because there was no increase in peptide-specific CTL with the use of rGM-CSF versus CTL responses induced by immunization with antigen alone [39]. Despite the lack of effect on antigen-specific CTL responses, rGM-CSF significantly increased the number of peptide-specific IFN*γ*-secreting cells as compared to responses induced by immunization with antigen alone (~150 versus ~10 per 10^6^ splenocytes, resp.) as well as the number of tetramer-positive CD8+ T cells (~1.5% versus <0.5%, resp.) [39]. Interestingly, similar to the results we reported for the addition of rIL-12 to the vaccine formulation, boosting all groups with antigen alone on day 42 significantly increased the effect of rGM-CSF on the induction of target cell lysis (~25%), increasing it to levels equal to those induced by CT (~30%) and IL-1*α* (~25%) (<5% by antigen alone). Ramsburg et al. also evaluated the ability of GM-CSF to increase CD8+ T-cell responses [62]. Unlike the other studies, however, they created an attenuated vesicular stomatitis virus (VSV) expressing GM-CSF. Compared to a virus expressing EGFP, GM-CSF increased the number of VSV tetramer-positive CD8+ T cells in the lungs (28.2% and 16.3%, resp.) 30 days postinfection. They also noted that VSV-GMCSF1 increased the ability of those T cells to expand after a booster immunization compared to VSV-EGFP.

### 7.4. Protection against Challenge

At least two studies have evaluated the ability of GM-CSF to increase protection against challenge. Similar to the work described by Ramsburg et al. with a GM-CSF-expressing VSV, Parker et al. constructed a GM-CSF-expressing HSV-1 and evaluated its ability to protect against challenge with 1 × 10^6^ pfu HSV [42]. The expression of GM-CSF by the virus induced HSV-1-specific immune responses that significantly increased survival compared to sham-immunized animals when mice were immunized with 10,000 or 100,000 pfu (22/38 and 39/39 surviving, resp.); immunization with 10,000 or 100,000 pfu of HSV-1 vaccine lacking GM-CSF induced survival of 12/39 and 34/40 mice, respectively. Compared to IL-12 (as discussed above), GM-CSF expression required higher immunization doses to induce protection. Nambiar et al. used a similar model and immunized mice with 5 × 10^5^ cfu BCG expressing GM-CSF and evaluated the bacterial load after an aerosol challenge with *M. tuberculosis *(unknown dose) [63]. Immunization with GM-CSG/BCG significantly decreased (~1 log reduction) the bacterial load in the spleen compared to immunization with noncytokine-secreting BCG when mice were challenged at 4 or 12 weeks following vaccination; lung CFUs were only decreased when mice were challenged at 4 weeks after immunization (~1.5 log reduction versus ~0.5 log reduction at 12 weeks).

## 8. IL-6

Although IL-6 is a potent inflammatory cytokine [64], it has also been shown to play a role in shaping adaptive immune responses as both a B-cell stimulating factor [64] and as a Th17-inducing cytokine [65].

### 8.1. Serum Antibody Production

At least two groups have evaluated the ability of IL-6 to augment serum antibody production following intranasal immunization. Following three immunizations with 10^9^ *L. lactococcus* expressing TTFC and IL-6, Steidler et al. demonstrated significantly increased production of serum anti-TTFC IgG by day 24 that persisted to at least day 80 when compared to serum anti-TTFC IgG titers induced by immunization with *L. lactococcus* expressing antigen alone (~1 log increase). Although the use of IL-6 as an adjuvant increased serum IgG titers, IL-6 expression did not appear to alter the profile of serum anti-TT IgG subclasses [23]. Additionally, serum anti-TTFC IgA production appeared to be increased by the inclusion of IL-6 compared to antigen alone; whether these differences were significant is unclear (~1 : 100 versus ~1 : 40 at day 80). Using a different model of delivery, IL-6-containing liposomes, Boyaka et al. also demonstrated significantly increased serum anti-TT IgG after three immunizations and a total of six 1 *μ*g IL-6 doses compared to antigen alone (~1 : 1,048,576 versus 1 : 4,096, resp.) [25]. This regimen also significantly increased serum anti-TT IgG1 and IgG2b over that induced by antigen alone (approx. 1 : 1,024 compared to 1 : 128 and 1 : 64, resp.), but it did not increase IgG2a or IgG3 production.

### 8.2. Mucosal Antibody Production

Boyaka et al. did not report increased mucosal IgA production when IL-6 was included in the vaccine formulation compared to TT alone [25]. However, they did observe a slight increase in the number of LPL TT-specific IgA AFCs induced by TT + IL-6 when compared to immunization with TT alone (140 and 23 AFCs, resp.). By contrast, Steidler et al. only investigated the impact of IL-6 on total mucosal IgA production after nasal immunization with TTFC and found no change when compared to mice immunized with TT alone. When Boyaka compared IL-6 to IL-12 after nasal immunization with TT [25], IL-12 was superior to IL-6 for inducing mucosal antibody production following vaccination with identical doses, as it significantly increased anti-TT IgA in fecal, vaginal, and saliva samples compared to TT alone; the number of anti-TT IgA AFCs was also increased, although it is unclear if this was significant. In addition to those two studies, Braciak et al. also evaluated mucosal IgA production following immunization with an adenovirus (Ad) expressing IL-6 [66]. Following immunization with 3 × 10^8^ pfu IL-6-expressing adenovirus, BAL anti-Ad IgA titers increased threefold over 14 days compared to baseline. Their more striking observation, however, was the increase in lung anti-Ad IgA ASCs at days 7 and 14 after immunization compared to adenovirus not expressing IL-6 (174 versus 38 per 10^6^ lymphocytes and 270 versus < 5 per 10^6^ lymphocytes, resp.). Given that two of the three studies observed increases in the number of antigen-specific IgA ASCs when using IL-6 as a nasal vaccine adjuvant, it may be that greater increases in mucosal IgA production could be seen if the IL-6 or antigen doses were increased in future studies.

### 8.3. Protection against Challenge

Following three immunizations with 75 *μ*g TT on days 0, 7, and 14 with rIL-6 delivered on days 0, 3, 7, 10, 14, and 17, Boyaka et al. observed increased protection against a systemic challenge with 100 MLDs of TT, with all mice surviving challenge compared to 0 mice immunized with TT alone [25].

## 9. Flt3 Ligand

Flt3 ligand (Flt3L) is expressed by stromal fibroblasts in the hematopoietic bone marrow environment and T lymphocytes, and it has been shown to have effects on the proliferation and differentiation of T cells, B cells, NK cells, and DCs [67].

### 9.1. Serum Antibody Production

Several studies have evaluated the nasal adjuvant activity of Flt3L, but the majority of these have been carried out by the same group at the University of Alabama, Birmingham [28, 29, 68] using OVA as the codelivered antigen. All three studies used the same immunization schedule (three times at weekly intervals) and antigen dose (100 *μ*g OVA), but one of the three delivered a plasmid expressing Flt3L (50 *μ*g) [28], the second delivered plasmids expressing CpG, Flt3L, or CpG plus Flt3L (50 *μ*g) [68], and the third used a Flt3L-expressing adenovirus (1 × 10^8^ pfu) [29]. When OVA was delivered with either pFlt3L or Ad-Flt3L, Kataoka et al. and Sekine et al. demonstrated serum anti-OVA IgG production that was significantly increased above that induced by OVA delivered with an empty vector (pFlt3L: 1 : 4,096 versus 1 : 524,288, resp.; Ad-Flt3L: 1 : 8,194 versus 1 : 131,072, resp.). Both studies also demonstrated increases in serum anti-OVA IgA production when Flt3L was coadministered with antigen compared to IgA responses induced by immunization with antigen alone, but only the study using the adenoviral vector noted the difference as significant (pFlt3L: 1 : 1,024 versus 1 : 64, resp., Ad-Flt3L: 1 : 2,048 versus <1 : 64, resp.). When pFlt3L was codelivered with pCpG and OVA, the serum anti-OVA antibody production was not significantly increased over pCpG + OVA or pFlt3L + OVA [68]. However, the anti-OVA IgG subclass profile did differ significantly, as OVA-specific IgG2a and IgG2b production (1 : 524,288) was significantly increased over that induced by CpG + OVA (1 : 65,536) or pFlt3L + OVA (1 :  32,768) and equal to the amounts of anti-OVA IgG1 induced by any formulation. Using the adenoviral vector, Sekine et al. also saw significant increases in anti-OVA IgG1, IgG2a, and IgG2b (1 : 8,192, 1 : 65,536, and 1 : 8,192, resp.) compared to an empty vector (1 : 1,024, 1 : 4,096, and 1 : 1,024, resp.). By contrast, Kataoka et al. did not note a significant increase in anti-OVA IgG2a following vaccination with OVA + pFlt3L, but they did see significantly increased anti-OVA IgG1 and IgG2b (1 : 262,144 and 1 : 16,384, resp., versus 1 : 2,048 and 1 : 1,024, resp., for antigen alone). Anti-OVA IgG3 was not increased in any of the studies.

In contrast to those studies, Kodama et al. used an entirely different immunization protocol [69]. In their study, 10 *μ*g rFlt3L was delivered alone on day 0 and antigen alone (*Haemophilus influenzae *P6) was delivered on days 6, 13, and 20. This protocol resulted in a significant increase in the number of NALT DCs prior to the first delivery of antigen. At one week following vaccination, serum anti-P6 IgG (1 : 131,072), IgM (1 : 64), and IgA (1 : 64) were significantly increased with the use of Flt3L when compared to those induced in mice immunized with P6 alone (1 : 128, undetectable, and undetectable, resp.). The increased serum titers decreased little through three months after vaccination in mice receiving P6 + Flt3L, but all serum titers were undetectable in mice given P6 alone. Taken together, these studies indicate that Flt3L can increase and maintain serum immune responses to codelivered antigen.

### 9.2. Mucosal Immunity

The four studies that investigated the ability of Flt3L to increase serum immune responses also evaluated its ability to increase mucosal immune responses [28, 29, 68, 69]. Two studies have demonstrated enhanced mucosal antibody production following vaccination with Flt3L. Following delivery of pFlt3L and antigen, Kataoka et al. significantly increased nasal wash and saliva anti-OVA IgA production (~1 : 128 and 1 : 23, resp.) over that induced by antigen alone (undetectable). Similarly, Sekine et al. demonstrated that OVA + Ad-Flt3L increased nasal wash and saliva anti-OVA IgA production (~1 : 32 and 1 : 16, resp.) compared to OVA + empty vector (~1 : 8 and 1 : 6, resp.). Both studies noted increases in the number of IgA anti-OVA AFCs at various mucosal sites, including the nasal passages (NPs), submandibular glands (SMGs), lamina propria (LP), and NALT when compared to anti-OVA IgA AFCs induced by immunization with OVA alone. Sekine et al. observed the largest adjuvant effect of Flt3L, with OVA alone generating few, if any, OVA-specific IgA AFCs while OVA + pFlt3L generated 100, 250, and 200 per 10^6^ cells in the NPs, SMGs, and LP, respectively. Kataoka et al. demonstrated increases to ~750, 50, and 40 anti-OVA IgA AFCs per 10^6^ cells in the NPs, SMGs, and NALT, respectively, with few, if any, induced by immunization with OVA alone. In contrast to these two studies, Fukuiwa did not note any significant increases in nasal or saliva anti-OVA IgA (1 : 32 and 1 : 64, resp.) or NP or SMG anti-OVA IgA AFCs (~180 and 200 per 10^6^ cells, resp.) when 50 *μ*g pFlt3L was delivered with 100 *μ*g OVA compared to OVA alone (undetectable) [68]. When Kodama et al. delivered 10 *μ*g rFlt3L on day 0 followed by three immunizations with 10 *μ*g of P6 antigen alone on days 6, 13, and 20, nasal anti-P6 titers were significantly increased by the addition of rFlt3L compared to immunization with antigen alone at both one week and three months after immunization (1 : 64 versus undetectable at both time points) [69]. Similarly, the number of anti-P6 IgA AFCs was significantly increased in the nasal passages at one week and three months after vaccination using rFlt3L when compared to IgA AFCs induced by immunization with antigen alone (~1,400 and 1,000, resp., versus ~250 per 10^6^ cells). However, they were not increased in the NALT. Therefore, similar to its ability to induce antigen-specific serum immune responses, Flt3L does appear to augment antigen-specific mucosal antibody responses as well. The differences in AFCs induced in the various mucosal sites investigated may be related to the methods in which Flt3L was administered, for example, plasmid, adenoviral vector, and recombinant protein.

### 9.3. Cell-Mediated Immunity

Two of the studies discussed above have evaluated the ability of Flt3L to increase cell-mediated immune responses to codelivered antigen. Following immunization with pFlt3L and OVA, Kataoka et al. demonstrated significantly increased splenocyte and CLN OVA-specific CD4+ T-cell proliferation seven days after the final immunization compared to OVA + empty vector [28]. Similarly, Sekine et al. evaluated splenocyte and CLN OVA-specific CTL activity seven days after the final immunization with Ad-Flt3L [29]. Compared to OVA plus an empty adenoviral vector, OVA + Ad-Flt3L significantly increased specific lysis in the CLNs and spleen at an E : T ratio of 25 : 1 (~30% and 75%, resp., versus 15% and 30% for OVA + Ad-luc, resp.).

### 9.4. Protection against Challenge

In their study, Kodama et al. evaluated the ability of Flt3L to enhance the clearance of 10^8^ cfu of a nasally delivered nontypeable *Haemophilus influenza *at one week and three months after the final immunization [69]. At both time points, the use of Flt3L as an adjuvant on day 0 of the immunization protocol significantly decreased the bacterial load in the nasal cavity by approximately 2 logs as compared to mice immunized with antigen alone.

## 10. IL-1

IL-1 is a potent proinflammatory cytokine with a wide range of effects on the host immune system. These effects include the up- and downregulation of many genes, including cytokine and chemokine receptors, cytokines and chemokines, and adhesion molecules, resulting in the trafficking of cell populations (e.g., neutrophils) into areas of inflammation [70]. IL-1 has also been shown to play a role in Th17 differentiation [71].

### 10.1. Serum Antibody Production

Our group was the first to use IL-1 as an adjuvant in nasally administered vaccines [72]. We have since used IL-1 with a variety of codelivered antigens in three animal models: mice [21, 31, 39, 72], rabbits [21], and monkeys [73]. In our first study, we codelivered 4 *μ*g of either IL-1*α* or IL-1*β* with 50 *μ*g TT or 100 *μ*g OVA and compared the induced immune responses to those induced by 1 *μ*g CT plus antigen. Both IL-1 proteins induced serum antigen-specific IgG equivalent to that induced by immunization with CT + antigen. We also evaluated the ability of CT and IL-1 to enhance serum antibody production when delivered with all three immunizations or only with the first immunization. Compared to TT delivered without adjuvant, IL-1 increased serum anti-TT IgG at least 2 log after delivery with all three immunizations and at least 1.5 log when delivered only with the first immunization [72]. When OVA was used as the antigen and the adjuvants were delivered with all three immunizations, both IL-1*α* and IL-1*β* increased serum anti-OVA IgG to approximately 1 : 100,000 compared to <1 : 1,000 for antigen alone and 1 : 10,000 for OVA + CT [72]. When IL-1 was only delivered with the first OVA immunization, it induced anti-OVA IgG titers of 1 : 10,000, while OVA alone and OVA + CT (delivered only with the first immunization) induced titers <1 : 1,000. In general, the anti-OVA IgG subclass profiles were similar between the mouse strains and adjuvants (i.e., IL-1*α*, IL-1*β*, and CT). However, when OVA was used as the antigen, IL-1 delivered with all three immunizations induced greater anti-OVA IgG1, IgG2a, and IgG2b responses than CT delivered with all three immunizations. In addition to protein antigens, we have also evaluated the ability of IL-1 to augment serum antigen-specific immune responses to an HIV peptide antigen [31]. Following three immunizations, codelivery of 4 *μ*g IL-1*α* increased antipeptide IgG production to ~1 : 50,000, compared to ~1 : 10,000 for antigen + CT and <1 : 10 for antigen alone. In a recently published study, we evaluated the ability of IL-1*β* to induce serum antibody responses to immunization with TT or pneumococcal surface protein A (PspA) [21]. When 5 *μ*g IL-1 was nasally codelivered with either PspA or TT, it induced serum antigen-specific IgG production equivalent to that induced by a subcutaneous injection of PspA with alum or an intranasal immunization with TT + CT. One other group has also evaluated the ability of nasally delivered IL-1 to augment antigen-specific serum antibody production [22]. Following immunization on days 0 and 28 with 1 *μ*g rHA plus 1 *μ*g of either IL-1*α* or IL-1*β*, Kayamuro et al. demonstrated significantly increased serum anti-HA IgG when compared to anti-HA IgG titers induced by immunization with rHA alone, though these responses were only reported as ODs at an undefined serum dilution. However, they also compared the IL-1-induced immune response to that induced by 1 *μ*g CT. At a serum dilution of 1 : 1,000, CT-induced anti-HA IgG was approximately 1.2 OD_450_, while IL-1*α* at 0.1–1 *μ*g increased anti-HA IgG to 0.7–1.1 OD_450_. In the absence of adjuvant, HA-induced responses were <0.2 OD_450_. Although it is difficult to compare the magnitude of the responses between their study and ours, it is evident from the inclusion of CT by both groups that IL-1 increased antigen-specific serum immune responses to levels equivalent to those induced by CT.

We have also evaluated the ability of nasally delivered IL-1 to augment immune responses in rabbits [21]. Unfortunately, the antigen-specific immune responses induced in the rabbits were highly variable and did not reflect a dose-responsive relationship with IL-1. When TT + IL-1 was delivered intramuscularly, the serum anti-TT IgG titer was significantly increased compared to the anti-TT IgG titer induced by intranasal delivery of TT + IL-1 (1 : 16,777,226 and 1 : 1,048,576, resp.).

### 10.2. Mucosal Antibody

Within the studies discussed above, we also evaluated the ability of IL-1 to induce mucosal antibody production. In our first study, 4 *μ*g IL-1*α* or IL-1*β* was codelivered with OVA or TT and compared to 1 *μ*g CT plus OVA or TT as well as antigen alone [72]. We compared the ability of the adjuvants to augment mucosal immune responses to the codelivered antigen when delivered only with the first immunization or with all three immunizations. Regardless of the codelivered antigen (TT or OVA), IL-1 was able to induce vaginal antigen-specific IgA when it was codelivered with antigen all three times (1 : 50–1 : 100), whereas CT was unable to induce vaginal anti-OVA IgA. CT was able to induce vaginal IgA production following vaccination with TT, but the increases were still less (not significantly) than those induced by TT + IL-1. In another experiment, following three immunizations with TT + IL-1*β*, doses from 0.2 to 5 *μ*g IL-1 were able to induce significantly increased vaginal anti-TT IgA (1 : 512–1 : 1,448), and the response was similar to that induced by 1 *μ*g CT (1 : 1,048). Kayamuro et al. have also demonstrated the ability of IL-1*α* and IL-1*β* to induce mucosal antibody production [22]. Following immunizations on days 0 and 28, they reported increased nasal wash anti-HA IgA following vaccination with HA + CT (1 *μ*g) or either IL-1 (0.2–1 *μ*g) at dilutions of 1 : 6 and 1 : 30 (OD_450_ ~ 1.2 and 0.6, resp.) compared to vaccination with HA alone (OD_450_ < 0.2). They also demonstrated significantly increased anti-HA IgA in saliva, vaginal, and fecal samples following immunization with HA + IL-1*α* or IL-1*β* as compared to immunization with HA alone, but these were reported in OD_450 _ at undefined sample dilutions. Although the different reporting methods make it difficult to directly compare the studies, IL-1 was shown by all studies described to increase mucosal immune responses to the codelivered antigen in mice. Despite the promising results in mice, IL-1 has not yet been shown to significantly enhance mucosal antigen-specific antibody production in rabbits. Although we have evaluated the ability of IL-1 to induce antigen-specific vaginal IgA production in rabbits, IL-1-adjuvanted vaccines did not induce IgA in rabbits under the conditions tested [21].

### 10.3. Cell-Mediated Immunity

In the studies discussed above, we also evaluated the ability of IL-1 to augment cell-mediated immune responses. In two studies in which we examined DTH following three intranasal immunizations, IL-1 significantly increased the antigen-specific ear swelling response to injected antigen over that induced by vaccination with antigen alone [21, 72]. Compared to CT, IL-1 induced significantly increased ear swelling following immunization with OVA [72]. However, when codelivered with TT, CT and IL-1 have been shown to induce similar degrees of ear swelling in both BALB/c and C57BL/6 mice [72]. 

We have also evaluated increases in antigen-specific lymphoproliferative responses following immunization with TT plus adjuvant [72]. IL-1*α*, IL-1*β*, and CT increased TT-specific lymphoproliferation to nearly 12,000 cpm in C57BL/6 and 16,000–22,000 cpm in BALB/c mice, while TT alone induced little lymphoproliferation. In addition, we have shown that IL-1*α* can significantly increase target cell lysis following four immunizations with 100 *μ*g HIV peptide and 4 *μ*g IL-1 [39]. Peptide-specific lysis at an E : T ratio of 25 : 1 was increased to 45% with the use of IL-1 as an adjuvant as compared to ~15% in mice immunized with antigen alone; this increase was similar to that induced by CT (~55%). In the same study, the number of antigen-specific IFN*γ*-secreting CD8+ T cells was also increased with the use of IL-1 compared to mice immunized with antigen alone (~150 per 10^6^ versus ~10 per 10^6^, resp.), while CT showed a similar increase to ~200 per 10^6^. The % of tetramer-positive CD8+ T cells was also increased (<0.5% versus 2.5% for antigen alone and antigen + IL-1, resp.). 

In contrast to the results we have reported, Kayamuro et al. evaluated the ability of IL-1*α* and IL-1*β* to induce HA tetramer-positive CD8+ T cells and peptide-specific IFN*γ*-secreting CD8+ T cells and failed to observe significant increases [22]. Although IL-1*α* did slightly increase the fluorescence intensity of tetramer-positive cells (~1.75 versus ~1.1 for HA alone), only CT significantly increased this response (~2.3). Similarly, HA + IL-1*α* or HA + IL-1*β* did not significantly increase the number of IFN*γ*-secreting CD8+ T cells compared to HA alone (35, 10, and 5 per 10^6^ splenocytes for IL-1*α*, IL-1*β*, and HA, resp.), although in this instance, neither did immunization with HA + CT (~25/10^6^ splenocytes) [22]. However, the variation in the response induced by IL-1*α* was relatively large. All of these studies indicate that IL-1 has the ability to augment cell-mediated immunity, though the magnitude of the induced response may differ based on the antigen or antigen dose used.

### 10.4. Protection against Challenge

We have evaluated the ability of IL-1 to provide adjuvant activity and enhance vaccine-induced survival following challenge with either 1.125 × 10^5^ cfu *S. pneumoniae* or 10 LD_50_ TT [21]. Intranasal immunization with PspA and IL-1 significantly increased survival following IV challenge on day 72 to 68% compared to 0% survival in mice immunized with PspA alone. This increase was equal to that induced by subcutaneous administration of PspA + alum (68%). Similarly, intranasal immunization with 0.2–5 *μ*g IL-1 + TT provided 100% protection following a TT challenge while immunization with TT alone provided only 10% protection. Kayamuro et al. also evaluated the ability of IL-1 to enhance vaccine-induced protection against challenge [22]. They intranasally administered 256 hemagglutinating units of influenza virus 14 days after the second of two immunizations (on days 0 and 28) and evaluated the ability of 1 *μ*g HA alone, HA + 1 *μ*g CT, or HA + 1 *μ*g IL-1*α* or IL-1*β* to protect against morbidity. While coadministration of CT with HA provided 100% survival, immunization with HA alone provided only 10% survival. The use of IL-1 as a nasal vaccine adjuvant provided significant protective immunity, as 80–85% of mice survived infection. Although in this model, IL-1 did not perform as well as CT, it did significantly increase survival following challenge.

## 11. Other Cytokines

Although this paper attempted to focus on some of the more well-studied cytokines, other cytokines and chemokines have been evaluated in one or two studies for their ability to augment antigen-specific immune responses following intranasal vaccination. Among these are RANTES [74, 75], TNF*α* [76], IFN*γ* [77], lymphotactin [78], IL-4 [77], IL-5 [66, 79], and IL-18 [22, 31, 39]. Although many of these have shown promise by inducing antigen-specific antibody responses above those induced by immunization with antigen alone, more studies are required to determine their real value as intranasally delivered vaccine adjuvants. For example, two studies have evaluated the nasal adjuvant activity of RANTES [74, 75] and have demonstrated its ability to augment serum and mucosal responses to codelivered OVA when given as a recombinant protein and cell-mediated immune responses to an HIV DNA vaccine as an expression plasmid. The same group that delivered rRANTES with OVA also evaluated lymphotactin and demonstrated its ability to increase serum anti-OVA IgG, IgA, and IgM at day 21 (one week after the final immunization) as well as mucosal anti-OVA IgA [78]. As another example, we have evaluated IL-18, an IL-1 family cytokine, for its ability to augment serum antibody production as well as cell-mediated immune responses. When 0.4 *μ*g IL-18 was delivered nasally with an HIV peptide, antipeptide IgG was significantly increased to levels equal to those induced by 1 *μ*g CT, 4 *μ*g IL-1*α*, and 4 *μ*g GM-CSF [31]. This dose was also able to significantly increase peptide-specific lysis and the number of IFN*γ*-secreting CD8+ T cells [39]. However, the induced cell-mediated immune responses were not as great as those induced by CT or IL-1*α*, indicating that a higher dose of IL-18 may be required for optimal responses. Although many different cytokines have been evaluated as nasal vaccine adjuvants, it is likely that they will not all be equally as good at generating all desired immune responses. More work will be required to evaluate the vaccine adjuvants mentioned to determine if they will in fact be effective at increasing antigen-specific immune responses to a variety of antigens in multiple animal models.

## 12. Cytokine Combinations

Although briefly mentioned above, cytokines have been used in combination with other cytokines or other vaccine adjuvants to induce antigen-specific immune responses. In several instances, the inclusion of a cytokine that may not have been effective by itself was enough to significantly increase immune responses when given with another adjuvant over those provided by either of them alone. In others, the combination of the cytokine adjuvants provided no increase in the immune response compared to just one delivered alone. The latter situation may reflect the activities of those particular cytokines. For example, two groups have shown that the combination of rIL-12 and rGM-CSF is a poor inducer of antigen-specific immunity [30, 39], and the combination of rIL-12 and rIL-6 was also found to be ineffective at increasing immune responses over those induced by IL-12 alone [25]. However, other cytokine combinations have shown more promise. We have previously shown that combinations of IL-1 + GM-CSF, IL-1 + IL-12, IL-1 + IL-18, and IL-1 + IL-12 + GM-CSF were significantly better inducers of cell-mediated immunity than these cytokines delivered individually [39]. We have also evaluated the ability of IL-1 + GM-CSF to augment antipeptide immune responses following intranasal immunization of macaques [73]. In this study, we demonstrated that IL-1 + GM-CSF induced antibody production in the nasal and genital mucosa following codelivery with an HIV peptide, while a mutant CT did not. However, parenteral delivery of GM-CSF in combination with an MPL analog (RC-529) and antigen elicited stronger responses than intranasal delivery. 

Two other groups have combined cytokine adjuvants with more common mucosal adjuvants, such as CTB and CpG [38, 68]. Using 1 *μ*g IL-12 and 10 *μ*g CTB codelivered with gp120, Albu et al. observed that only the combination of the two increased immune responses over those induced by antigen alone or antigen with either IL-12 or CTB [38]. Similarly, although both CpG and pFlt3L alone induced increases in antigen-specific immune responses, Fukuiwa et al. demonstrated that the combination of the two induced more potent mucosal IgA production and serum anti-OVA IgG2a and IgG2b than either of them delivered alone [68]. As many noncytokine adjuvant combinations have recently been evaluated (e.g., GlaxoSmithKline's adjuvant systems) and the combination of alum and MPL has recently received FDA approval for parenteral delivery [80], more in-depth studies will be valuable in determining just how combinations of cytokine adjuvants will be useful for increasing specific immune responses in multiple animal models.

## 13. Safety of Nasally Administered Vaccines

Toxins and toxin derivatives are not the only current safety concern for nasal vaccines. Many groups have expressed concern about the potential for nasal vaccines to result in the delivery of nasal vaccine antigens or adjuvants into the brain. Several studies have examined the ability of various compounds to enter the brain after nasal delivery and have compared nasal to systemic routes of immunization. Although many compounds, such as interferon, have the ability to enter the olfactory bulb and the brain after nasal delivery [81], few side effects were observed following nasal immunization with an aerosolized measles vaccine lacking toxin adjuvants in humans [82]. Additionally, the live-attenuated, nasally administered influenza vaccine, FluMist, has been approved by the FDA and is considered safe [83]. Although the significance of enterotoxin delivery or redirection of vaccine antigens to the olfactory bulb is unclear, it is evident from the development of Bell's palsy following vaccination with mutant LT that safer mucosal vaccine adjuvants are required for use in human vaccines[16]. Additional studies are needed to carefully evaluate local and systemic toxicity after nasal immunization using cytokine adjuvants.

## 14. Conclusions

As demonstrated in this paper, many cytokines have been evaluated as adjuvants for their ability to augment antigen-specific immune responses following nasal delivery. It is evident, however, that more work is required before cytokine adjuvants can be said definitively to be potent vaccine adjuvants for any of the responses discussed above. It is important to always consider the methods of antigen and adjuvant delivery (e.g., recombinant proteins, plasmids) as well as the types of antigen being used when evaluating any adjuvants for their effectiveness. Nonetheless, cytokines have so far shown promising results when given nasally with a variety of vaccine antigens and have the ability to induce many different types of immune responses. As such, cytokines may be ideal for inclusion in vaccines in which only certain immune responses are desired. In the future, more studies need to evaluate cytokine adjuvants with respect to other well-known vaccine adjuvants, such as CT or TLR ligands, to give study results more meaning with respect to the already published literature.

## Figures and Tables

**Table 1:** Cytokines enhance antigen-specific immunity following nasal delivery with antigen in mice.

Cytokine			Serum antigen-specific IgG		Mucosal antigen-specific IgA		Antigen-specific cell lysis		Survival following challenge		
	Reference	Ag	Ag alone	Ag + Cytokine	Ag alone	Ag + Cytokine	Ag alone	Ag + Cytokine	Ag	Ag alone	Ag + Cytokine
IL-1	[21] [22]	TT PspA HA	1 : 1,176 <1 : 32 <0.05^a^	1 : 356,722 1 : 50,766 0.4^a^	V: <1 : 16 V: <1 : 16 N: <0.2^b^	V: 1,448 V: 1 : 37 N: 0.6^b^	N/A	N/A	TT *S. pneumoniae * H1N1	10% 0% 10%	100% 68% 80–85%

IL-2	[23] [24]	TTFC *ca* Sing	1 : 10,000 N/A	1 : 100,000 N/A	N/A N: 1 : 50	N/A N: 1 : 200	N/A	N/A	N/A H2N2	N/A 57%	N/A 100%

IL-6	[25]	TT	1 : 4,096	1 : 1,048,576	V: 1 : 4	V: 1 : 4	N/A	N/A	TT	0%	100%

IL-12	[26] [25]	BHV gD TT	Undetect. 1 : 4,096	1 : 75,000 1 : 2,097,152	L: 1 : 66 V: 1 : 4	L: 1 : 577 V: 1 : 23	N/A	N/A	N/A TT	N/A 0%	N/A 100%

IL-15	[27]	gBDNA	1 : 19,061	1 : 22,000	V: 1 : 126	V: 1 : 505	~35%	~42%	HSV	33%	83%

Flt3L	[28] [29]	OVA Ad^c^	1 : 4,096 1 : 8,194	1 : 524,288 1 : 131,072	N: 1 : 4 N: 1 : 8	N: 1 : 128 N: 1 : 32	N/A 30%	N/A 75%	N/A	N/A	N/A

GM-CSF	[30] [31]	HIV DNA HIV^ d^	1 : 4,000 <1 : 10	1 : 16,000 1 : 10,000	F: 1 : 5,700 N/A	F: 1 : 16,000 N/A	N/A	N/A	N/A	N/A	N/A

MIP-1*α*	[32]	OVA	1 : 128	1 : 131,072	N: <1 : 2	N: 1 : 9	0%	50%	N/A	N/A	N/A

Type I IFN	[33] [34]	HA HA	1 : 200 1 : 10,000	1 : 35,000 1 : 316,000	N: 1 : 100 B: 1 : 630	N: 1 : 300 B: 1 : 6,300	N/A	N/A	H1N1 H1N1	60% 40%	100% 100%

^a^Indicates OD_450_ at a dilution of 1 : 5000.

^b^Indicates OD_450_ at a dilution of 1 : 30.

^c^Adenovirus.

^d^HIV C4-V3 MN peptide.

N: nasal lavage; V: vaginal lavage; F: fecal extract: B: bronchoalveolar lavage.
